# Cases Observed

**Published:** 1847-07

**Authors:** S. Henry Dickson

**Affiliations:** Prof. Instit. and Pract. Med. Col. State So. Ca.


*Cases observed by* S. HENRY DICKSON, M. D., Prof. Instit. and Pract. Med. Col. State So. Ca.—The habit of keeping a case-book is one which should be recommended to every physician commencing practice. Such a record is advantageous to himself, in a great diversity of modes, and may be made interesting and instructive to others. A *fact* clearly stated can scarcely fail to possess some importance, absolute or relative. On looking back upon a collection of professional memoranda, I have thought an occasional extract might, at least, entertain, for a moment, the readers of the Southern Journal of Medicine, &c., and offer for publication the following, among the first which presented themselves on turning over the leaves.

1. *Hydrophobia.*—On the 5th February, 1840, I was desired to visit Julius, a black boy, three years of age, belonging to Mrs. M. He had been sick since the evening of the day before; had complained of pain in his head and stomach; had taken a purgative and voided a worm, (*lumbricus.*) His skin was cool; his pulse very frequent; his lips slightly livid; his tongue of natural appearance. His eye was wild; his countenance anxious; and he sighed and sobbed a good deal; he said his head and stomach hurt him, but not much.

Confessing that I did not clearly comprehend the case, I advised that he should be put into the warm bath, and a mustard poultice afterwards applied over the abdomen, and prepared for him a mixture of carb. potass. with tinct. opii. camph. This was in the afternoon.

Next morning, (6th,) his mistress who had attended to him assiduously, mentioned that there was something very peculiar in his manner of taking his medicine, which alarmed her; and stated that he had been, some months before, bitten by a dog. On looking at his arm, we found the scars, four in number, quite noticeable, projecting slightly. His pulse was now feeble, and even more frequent than yesterday; he shewed incessant fear of something undefined, and an anxious desire of change of place, throwing his limbs about carelessly and irregularly, in a manner resembling the movements in chorea; the skin was cold; the lips livid; the eye staring, and the pupil so widely dilated that the iris seemed a mere ring.

I blew suddenly upon his cheek, and was startled by the horrid convulsion into which he was thrown—indeed, it seemed likely to prove immediately fatal. A few drops of water, sprinkled on him, produced a similar effect. He was dreadfully agitated at the sight of water, and refused to drink anything. His breathing was little more than a mere succession of sobs. He still walked, though with a staggering step and great debility.

A few hours elapsing, he became thirsty, and asked for water, which he grasped at with frantic eagerness, throwing his arms about; he succeeded in swallowing some of it, in spite of the violent spasm brought on by the attempt, menacing prompt suffocation. Indeed, he now seemed more convulsed when a puff of air was blown at him, or a few drops of water sprinkled on him, than when he made the effort to drink. Terror was strongly depicted in his countenance; his muscles were scarcely a moment at rest; he became very loquacious, complaining of headache and great thirst, and often calling for water. He died in the evening, having been ill about fifty-four hours.

A post-mortem examination shewed the membranes of the brain much injected, while the cerebral substance had undergone almost no change in appearance. There was nothing of morbid alteration discoverable in the viscera of the thorax or abdomen. There was well-marked subcutaneous inflammation beneath and around the scars on his arm, left by the bite from which he suffered.

This case is instructive, from the age and condition of the subject. The phenomena were purely physical, and, of necessity, free from any intermixture of imaginative conditions. The dog which inflicted the wounds had not been thought diseased, but was killed on the spot, as a worthless little animal, by his owner, whom he vexed by attacking him also. The wounds were very slight, and healed quickly, having excited no apprehension whatever. The occurrence took place in the September previous—an interval of between four and five months separating the injury from its fatal consequences.

The most impressive symptom here was the super-sensitiveness of the cutaneous surface. This was, in degree, extreme, and singularly characteristic, as it seemed most readily to respond to the impulse of atmospheric air set in motion. A sudden touch would, indeed, startle the patient, and the attempt to swallow would threaten suffocation; but, a puff of breath from the mouth, or the sensation communicated by waving the hand towards him, would bring on spasm and convulsion.

This peculiar symptom presented itself also, very strikingly, in a second case, which I saw some months after, with Dr. Bellinger. His patient, too, was young and unimaginative—a fine, intelligent boy, of about 11 years of age, son of respectable Irish parents. He died in August, having been bitten in April; never suspecting, so far as I am aware, any connection between his attack and the injury received so long before. The whole history of this case, indeed, strongly resembled that of which I have above given a brief statement.

2. *Bifid Vagina.*—Mrs. —— came to the city, 1839, to consult me. She has been two years married—has always suffered from irregular and scanty menstruation; it is but a few months since she has become aware of the existence of some genital malformation. The vagina is divided—neither longitudinally, nor transversely, but obliquely—by a membranous partition. Both tubes are long and narrow. Coition is difficult, particularly if the right (and somewhat anterior) opening be entered. The left, which is obliquely posterior, leads to the uterus, the os tincæ presenting; the right conducts to the side of the uterus in which the membraneous partition loses itself; the cul de sac is not to be reached by the finger; a long probe or bougie may pass up six inches or more, but gives pain, and, when withdrawn, is coated with bloody mucus. The dividing membrane lies in loose folds; is smooth and well lubricated; projects slightly between the labia. It possesses very little sensibility.

3. *Obliteration of the Vagina.*—I have seen three instances of this condition of the female parts. One was in an old African woman, who could give no history of the affair at all. She had, strangely enough, a stone in the bladder, the irritation of which gave the first occasion for discovering the fact. There was a very small opening, just sufficient to allow the insertion of a probe, which it was necessary to introduce frequently, to remove small urinary concretions or calculi, which presented themselves. She was upwards of ninety years old when I first saw her, and lived to be near a hundred—and, of course, as she was always relieved by the probe, was not a subject for lithotomy.

The second was in an old lady, often attacked by suppression of urine, but free from any calculous deposit. The labia had, in this case, as in the preceding, united so perfectly that there was not left any trace of a vulva, except a mere elevated ridge—a sort of raphe—in the perineum. The opening which communicated with the bladder only admitted a probe or very small bougie. She had suffered from difficult labor, in which the parts had undergone much injury, from unskilful handling, followed by long and severe inflammation.

The third was a most unfortunate instance, resulting, in a beautiful young married woman, from inflammation of the womb and its appendages, after a protracted first labour. The obstruction here occurred about an inch and a half within the vagina, and was exceedingly firm and unyielding to the touch, seeming fibro-cartilaginous. I advised waiting till menstruation should be attempted, in the hope that the projection of some portion of the obstructing tissue might direct where an opening could be made with safety. But, although there are sufficient reasons to believe that the ovaria did not partake in the disorganization and derangement inflicted, yet years have elapsed without any such effort as I have alluded to.—*Southern Journ. of Med. and Pharm.*

